# *Coptidis Rhizoma* Extract Reverses 5-Fluorouracil Resistance in HCT116 Human Colorectal Cancer Cells via Modulation of Thymidylate Synthase

**DOI:** 10.3390/molecules26071856

**Published:** 2021-03-25

**Authors:** Yong-Hwi Kang, Jin-Seok Lee, Nam-Hun Lee, Seung-Hyung Kim, Chang-Seob Seo, Chang-Gue Son

**Affiliations:** 1 Institute of Bioscience & Integrative Medicine, Daejeon Oriental Hospital of Daejeon University, Daeduk-daero, Seo-gu, Daejeon 35353, Korea; pig6315@naver.com (Y.-H.K.); neptune@dju.kr (J.-S.L.); 2 Department of Clinical Oncology, Cheonan Oriental Hospital of Daejeon University, 4, Notaesan-ro, Seobuk-gu, Cheonan-si 31099, Korea; 3 Institute of Traditional Medicine & Bioscience, Daejeon University, Daehak-ro 62, Dong-gu, Daejeon 34520, Korea; sksh518@dju.ac.kr; 4 Research Infrastructure Team, Herbal Medicine Research Division, Korea Institute of Oriental Medicine, 1672 Yuseong-daero, Yuseong-gu, Daejeon 34054, Korea; csseo0914@kiom.re.kr

**Keywords:** colorectal cancer, chemoresistance, 5-fluorouracil, thymidylate synthase, *Coptidis rhizoma*

## Abstract

Colorectal cancer (CRC) is a malignancy of the colon or rectum. It is ranked as the third most common cancer in both men and women worldwide. Early resection permitted by early detection is the best treatment, and chemotherapy is another main treatment, particularly for patients with advanced CRC. A well-known thymidylate synthase (TS) inhibitor, 5-fluorouracil (5-FU), is frequently prescribed to CRC patients; however, drug resistance is a critical limitation of its clinical application. Based on the hypothesis that *Coptidis Rhizoma* extract (CRE) can abolish this 5-FU resistance, we explored the efficacy and underlying mechanisms of CRE in 5-FU-resistant (HCT116/R) and parental HCT116 (HCT116/WT) cells. Compared to treatment with 5-FU alone, combination treatment with CRE and 5-FU drastically reduced the viability of HCT116/R cells. The cell cycle distribution assay showed significant induction of the G0/G1 phase arrest by co-treatment with CRE and 5-FU. In addition, the combination of CRE and 5-FU notably suppressed the activity of TS, which was overexpressed in HCT116/R cells, as compared to HCT116/WT cells. Our findings support the potential of CRE as an adjuvant agent against 5-FU-resistant colorectal cancers and indicate that the underlying mechanisms might involve inhibition of TS expression.

## 1. Introduction

Colorectal cancer (CRC) is the most common malignancy among various cancer types. In 2020, approximately 1.9 million people were diagnosed with CRC, and 935,000 deaths from CRC occurred worldwide [1]. The World Health Organization (WHO) estimates that the numbers of patients with CRC and CRC-related deaths will increase by 77 and 80% by 2030 [2]. The high mortality rate of CRC patients results from late diagnosis, which is due to the lack of clear physical symptoms at early stages [3]. Early resection, as permitted by early detection, is optimal CRC treatment. However, patients with advanced CRC mainly depend on chemotherapy [4].

One meta-analysis reported high expression of thymidylate synthase (TS) in the majority of CRC patients and an inverse association of TS expression with overall survival [5]. 5-Fluorouracil (5-FU), a potent inhibitor of TS, is an analogue of uracil, which has a fluorine atom instead of hydrogen at the C-5 position, which quickly enters cells using an identical facilitated transport mechanism like uracil. 5-FU is converted intracellularly to metabolites like fluorouridine triphosphate (FUTP), fluorodeoxyuridine triphosphate (FdUTP), and fluorodeoxyuridine monophosphate (FdUMP). These active metabolites interfere with DNA synthesis by TS [6]. It is commonly prescribed for patients with advanced or inoperable metastatic CRC [7,8]. Its beneficial outcomes as both a first-line chemotherapeutic treatment for primary colon tumors [9] and a palliative chemotherapy for metastatic cancer [10] were reported. However, the critical limitation of 5-FU is the development of drug resistance in tumor cells after long-term exposure to 5-FU [11]. The strategies to overcome 5-FU resistance are to increase the dose of 5-FU or to combine it with other anticancer drugs [12,13]. Increasing the dose of 5-FU exacerbates adverse effects, including general symptoms like bone marrow suppression, diarrhea, nausea, and vomiting [14,15,16,17,18], while combining it with other agents like oxaliplatin and leucovorin showed a limited response rate of 50% in CRC [17].

On the other hand, medicinal herbs, like *Coptidis Rhizoma* (*C. Rhizoma*), *Fructus Evodiae*, *Andrographis paniculata*, *Salvia miltiorrhiza*, *Hedyotis diffusa*, *Sophora flavescens*, *Curcuma longa* and *Bufo gargarizans*, recently attracted a great deal of attention as alternative anticancer therapies to reduce drug resistance [4,18]. Among these medicinal herbs, our group devoted attention to *C. Rhizoma*, which was used to treat bacillary dysentery, diabetes, pertussis, sore throat, naphtha, and eczema in traditional Chinese/Korean medicine for thousands of years [19]. Moreover, recent studies reported its pharmaceutical properties, including proliferation suppression, cell cycle arrest, and inhibition of tumor growth and inflammation, in vitro and in vivo [20,21,22].

In this study, we investigated the possible effect of *C. Rhizoma* against reversing 5-FU resistance in CRC and explored its underlying mechanisms using the HCT116/5-FU cell line.

## 2. Results

### 2.1. Compounds Present in CRE

**Figure 1:**
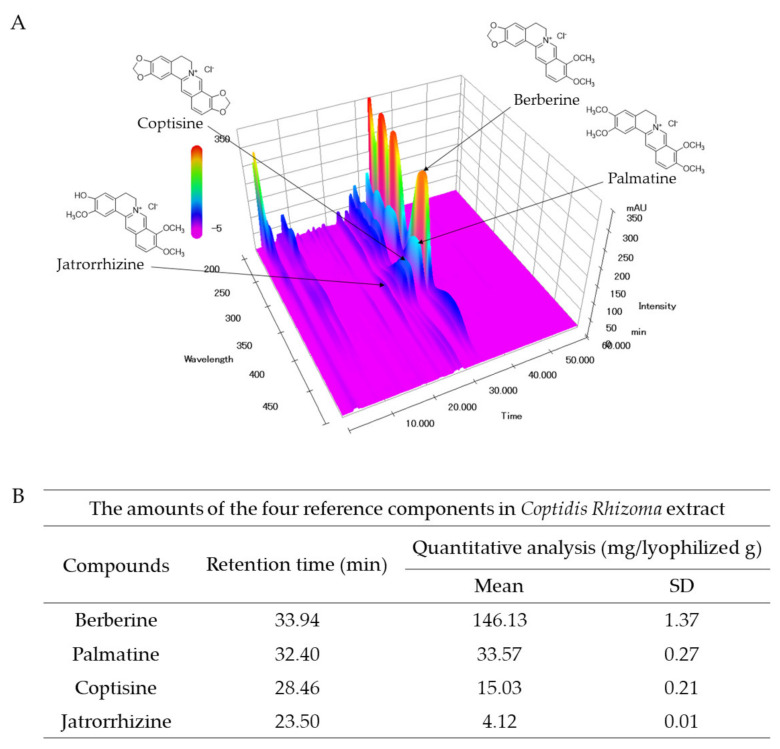
Fingerprint analysis of *Coptidis Rhizoma* extract (CRE). CRE and the four reference compounds were subjected to HPLC–PDA, and a 3D chromatogram was obtained at wavelengths of 200 to 500 nm (**A**). The amounts of the four major compounds, jatrorrhizine, coptisine, palmatine, and berberine, in CRE were quantified (**B**).

In the fingerprint analysis of CRE, the reference peaks were detected at retention times of 23.50 (jatrorrhizine), 28.46 (coptisine), 32.40 (palmatine), and 33.94 (berberine) min at a UV wavelength of 265 nm. The amount of the four reference compounds were quantified as follows—4.12 (jatrorrhizine), 15.03 (coptisine), 33.57 (palmatine), and 146.13 (berberine) mg/lyophilized g of CRE (Figure 1A,B).

### 2.2. Characteristics of HCT116/R Cells

**Figure 2:**
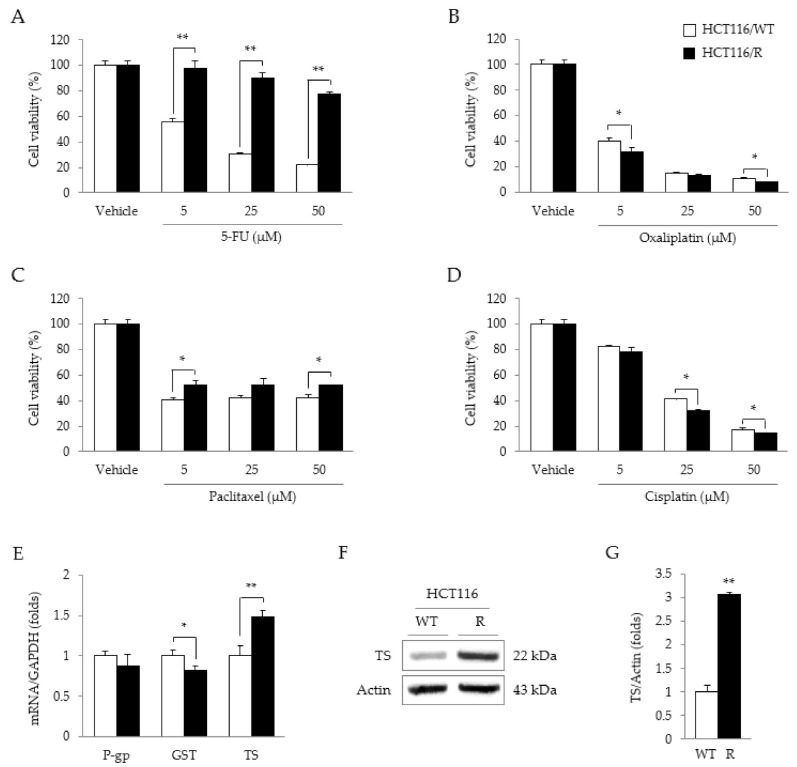
5-FU specific resistance is mediated by TS overexpression. Cell viability was measured to investigate anticancer drug resistance against 5-FU (**A**), oxaliplatin (**B**), paclitaxel (**C**), and cisplatin (**D**), in both HCT116/WT and HCT116/R cells. Gene expression levels of P-gp, GST, and TS were analyzed in HCT116/WT and HCT116/R cells (**E**). Protein expression levels (**F**) of TS and their quantitative comparisons (**G**) between HCT116/WT and HCT116/R cells were analyzed. Data are expressed as the mean ± SD (*n* = 3) values. * *p* < 0.05 and ** *p* < 0.01, as compared to the vehicle-treated cells or the corresponding parental cells.

Compared to HCT116/WT cells, HCT116/R cells showed marked resistance to 5-FU-induced cytotoxicity (*p* < 0.01 for all doses—5, 25, and 50 µM, Figure 2A). However, this pattern was not observed for the other three anticancer drugs (oxaliplatin, paclitaxel, and cisplatin) (Figure 2B–D). In addition, TS mRNA (but not P-gp or GST mRNA) was significantly overexpressed in HCT116/R cells, as compared to the parental cells (*p* < 0.01, Figure 2E). The TS protein was also markedly overexpressed by 3-fold in HCT116/R cells as compared to HCT116/WT cells (*p* < 0.01, Figure 2F,G).

### 2.3. Synergistic Effects of 5-FU and CRE on Properties Related to 5-FU Resistance

**Figure 3:**
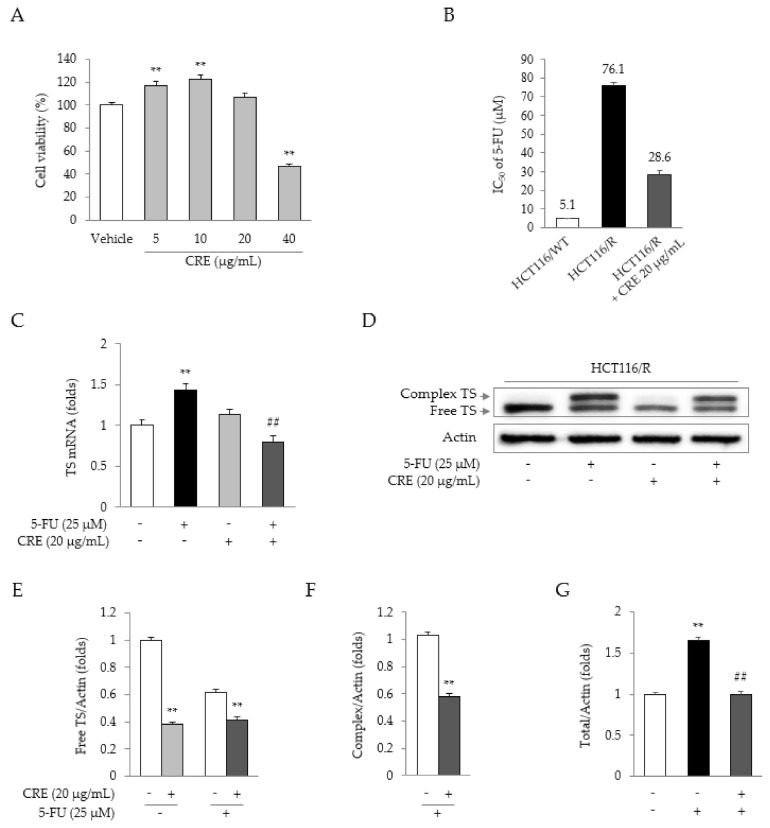
CRE reverses drug resistance by downregulating TS expression. HCT116/R cells were treated with various concentrations of CRE (**A**). Anti-proliferative IC_50_ value of co-treatment with CRE or 5-FU in HCT116/WT and HCT116/R cells (**B**). TS gene expression was measured in HCT116/R cells treated with CRE or 5-FU (**C**). The protein level of TS was measured in HCT116/R cells treated with CRE or 5-FU (**D**), and the intensities of free (**E**), complexed (**F**), and total TS (**G**) were quantified. Data are expressed as the mean ± SD (*n* = 3) values. ** *p* < 0.01, as compared to vehicle-treated cells. ## *p* < 0.01 compared to 5-FU-treated cells.

Treatment with CRE (up to 20 μg/mL) did not reduce the proliferation of HCT116/R cells (Figure 3A). However, compared to 5-FU treatment alone, combination treatment with 5-FU and CRE (20 μg/mL) significantly reduced the IC_50_ of 5-FU (Figure 3B). Treatment with 5-FU significantly upregulated TS gene expression, whereas this upregulation was notably attenuated by co-treatment with CRE (*p* < 0.01, Figure 3C). Treatment with CRE notably reduced the production of both free TS and the classical complex compared to that in cells not treated with CRE (*p* < 0.01, Figure 3D–G).

### 2.4. Synergistic Effects of 5-FU and CRE on Cell Cycle Arrest

**Figure 4:**
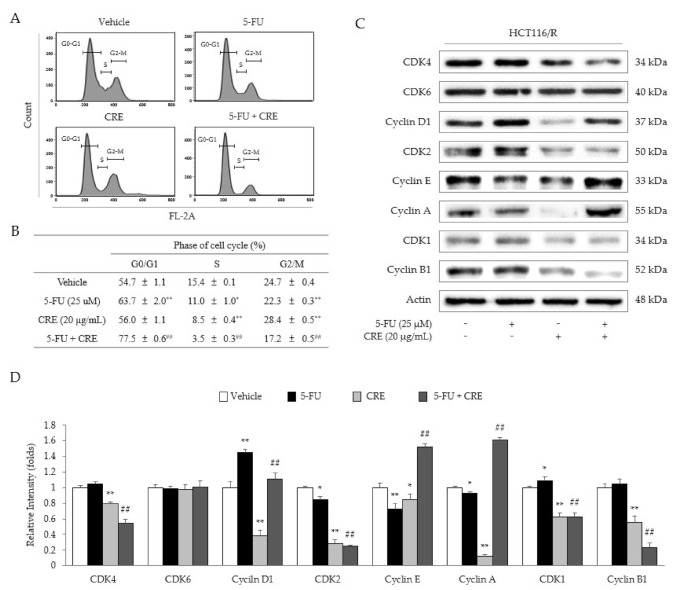
Cell cycle arrest is induced by co-treatment with 5-FU and CRE. The cell cycle distribution was analyzed in HCT116/R cells treated with CRE or 5-FU (**A**), and the relative percentages are indicated (**B**). Cell cycle-related protein expression levels (**C**) and the corresponding intensities were quantified (**D**). Data are expressed as the mean ± SD (*n* = 3) values. * *p* < 0.05 and ** *p* < 0.01, as compared to vehicle-treated cells. ## *p* < 0.01 compared to 5-FU-treated HCT116/R cells.

In HCT116/R cells, 5-FU treatment increased the proportion of G0/G1-phase cells (along with significant decreases in the S- and G2/M-phase cells), as compared to that in the vehicle group, while this increase was significantly enhanced by co-treatment with CRE (20 μg/mL, *p* < 0.01; Figure 4A,B) as compared to treatment with 5-FU alone. Treatment with CRE alone decreased the population of G2/M-phase cells (*p* < 0.01) compared to vehicle treatment (Figure 4A,B). These results were partially supported by analyses of cell cycle-related proteins, especially the notable alterations in cyclins B and D as well as CDK1, 2, and 4 (*p* < 0.05 or *p* <0.01, Figure 4C,D).

### 2.5. Identification of the Active Compound in CRE

**Figure 5:**
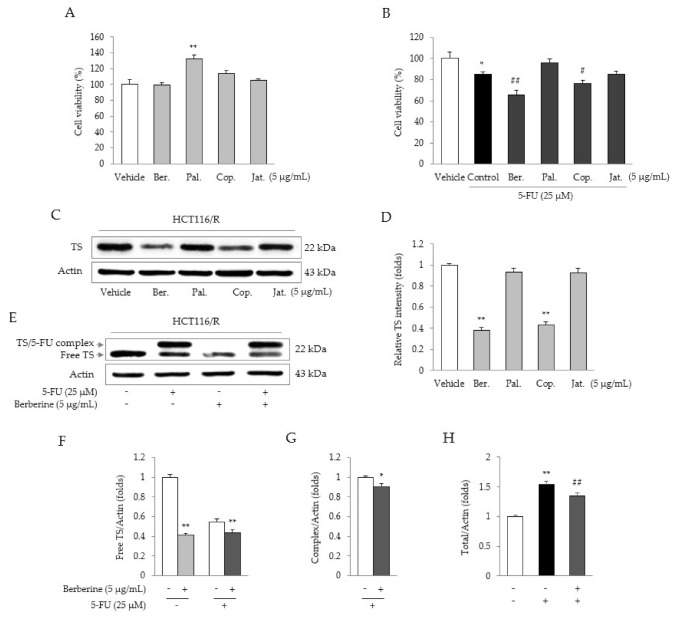
Investigation to identify the active compound of CRE. Cell viability was measured by treatment with the four main compounds of CRE (**A**) and evaluation of their synergistic effects with 5-FU in HCT116/R cells (**B**). TS protein expression levels (**C**) and their quantification (**D**) were determined in HCT116/R cells. The effects of berberine on TS protein expression were determined with/without 5-FU treatment (**E**), and the intensities of free (**F**), complexed (**G**), and total TS (**H**) were quantified. Data are expressed as the mean ± SD (*n* = 3) values. * *p* < 0.05 and ** *p* < 0.01 compared with vehicle-treated cells. # *p* < 0.05 and ## *p* < 0.01 compared with 5-FU-treated cells. Ber; berberine, Pal; palmatine, Cop; coptisine, Jat; jatrorrhizine

The four main compounds in CRE (5 μg/mL berberine, palmatine, coptisine, and jatrorrhizine) did not show any cytotoxicity in HCT116/R cells (Figure 5A). Moreover, co-treatment with berberine (5 μg/mL, *p* < 0.05) or coptisine (5 μg/mL, *p* < 0.05) significantly reduced cell viability, as compared to 5-FU treatment alone (Figure 5B). Both berberine and coptisine (but more potently berberine) significantly suppressed the protein expression of TS (*p* < 0.01, Figure 5C,D). Furthermore, berberine notably reduced TS protein production (*p* < 0.05 or *p* < 0.01) under both untreated and 5-FU-treated conditions (Figure 5E–H).

## 3. Discussion

5-FU is employed as a first-line therapy for patients with CRC [23]. However, during the 5-FU-based chemotherapy, resistance to 5-FU develops during 5-FU-based chemotherapy in more than 40% of patients with advanced CRC [24]. In fact, drug resistance is a typical hurdle in clinical oncology, including in CRC [25]. This resistance decreases the beneficial drug response but increases adverse effects because of the high dose of the anticancer drug [26]. In this study, we investigated the anti-5-FU resistance effects of CRE using HCT116/R cells with 5-FU-specific resistance.

As expected, HCT116/R cells showed significant enhancement of behaviors indicating resistance to the antiproliferative effects of two TS inhibitor (5-FU, Raltitrexed) compared to wild-type HCT116 cells (Figure 2A and Figure S1B), while these resistance behaviors were significantly attenuated by co-treatment with 5-FU and CRE (Figure 3B). This effect was not caused by cell death, as evidenced by no notable release of intracellular LDH to outside the cell (Figure S1A). It might be due to anti-proliferative effects of CRE when it does act with 5-FU as synergistic effects. HCT116/R cells were notably resistant to only 5-FU and not to oxaliplatin, paclitaxel, or cisplatin (Figure 2B–D). One previous study reported that *C. Rhizoma* reversed oxaliplatin resistance in HCT116 cells via modulation of P-gp [18]. P-gp and GST are considered the most common molecules involved in chemotherapeutic resistance [27], but the HCT116/R cells adapted in our study did not overexpress P-gp or GST (Figure 2E).

5-FU, a typical inhibitor of TS, plays a key role in thymidine synthesis, leading to DNA duplication and cell growth [28]. HCT116/R cells showed the overexpression of TS at both the mRNA and protein levels (Figure 2E–G), which was significantly attenuated by co-treatment with 5-FU and CRE (Figure 3C–G). A previous study presented an inverse relationship between the TS expression level and clinical outcomes, including overall survival and the response rate to 5-FU treatment in CRC patients with metastases [5,29]. These results indicate the possible identity of CRE as an agent for overcoming TS-mediated 5-FU resistance. In fact, the present data showed that CRE alone could suppress TS expression at both the gene and protein levels (Figure 3C,D).

The TS-mediated anti-5-FU resistance effect of CRE was partially supported by the cell cycle analysis results. A previous study showed that 5-FU treatment induced G0/G1 phase arrest in CRC [30]. Then, this 5-FU-induced G0/G1 phase arrest was significantly augmented by co-treatment with CRE (Figure 4A,B). The cell division cycle comprises the sequential events necessary for DNA replication and the production of two daughter cells. This process was regulated according to both the intracellular and extracellular status and depended on two key classes of regulatory molecules, cyclins, and CDKs [31]. In this study, co-treatment with 5-FU and CRE significantly decreased the levels of G1-specific CDKs (CDK2 and 4), while cyclins for the DNA synthesis phase (cyclins A and E) and G2 phase (cyclin A) were over expressed (Figure 4C,D). In fact, CRE alone also suppressed most cell cycle-related proteins. Accordingly, co-treatment (5-FU and CRE)-induced G0/G1 phase arrest was not clearly distinguished in the present study. Among the current results, suppression of CDK 4 was the most prominent change after co-treatment with 5-FU and CRE. A CDK4/6 inhibitor (palbociclib) showed a synergistic effect with irinotecan against colorectal cancer [32].

*C. Rhizoma* contains various pharmacological compounds, such as berberine, palmatine, coptisine, and jatrorrhizine [33,34]. Among these compounds, berberine (5 μg/mL) most significantly decreased the viability of HCT116/R cells under treatment with 5-FU (Figure 5B). Furthermore, this co-treatment significantly decreased the expression of free TS protein (Figure 5E,F). Although the efficacy of coptisine (5 μg/mL) was similar to (but slightly lower than) that of berberine (5 μg/mL), its compositional abundance was approximately 0.1-fold that of berberine (15.03 ± 0.21 versus 146.13 ± 1.37, Figure 1B). A previous study also reported that berberine induces cell cycle arrest in G0/G1 phase [35]. These data suggest that berberine is the active compound responsible for the anti-5-FU resistance effect of CRE.

## 4. Materials and Methods

### 4.1. Preparation of C. Rhizoma Extract (CRE)

*C. Rhizoma* (*Coptis chinensis*) was purchased from the Jeong-Seong Traditional Medicine Company (Daejeon, Korea). One hundred grams of *C. Rhizoma* was finely ground and extracted in 1 L of 30% ethanol for 72 h at room temperature, and the supernatants were then filtered through Whatman filter paper (Advantec^®^, Tokyo, Japan). The filtrate was concentrated in a rotary evaporator and lyophilized, and the final extraction yield was 5.5% (*w/w*).

### 4.2. Chemicals and Reagents

RPMI 1640 medium, fetal bovine serum (FBS), Dulbecco’s phosphate-buffered saline (DPBS), penicillin-streptomycin solution, and trypsin-ethylenediaminetetraacetic acid (EDTA) (WELGENE, Daegu, Korea); thymidylate synthase (TS, Cell Signaling Technology, Danvers, MA, USA); cyclin-dependent kinase (CDK1), cyclin-dependent kinase 2 (CDK2), cyclin-dependent kinase 4 (CDK4), cyclin-dependent kinase 6 (CDK6), cyclin A, cyclin B1, cyclin D1, cyclin E (Santa Cruz Biotechnology, Dallas, TX, USA); β-actin (Thermo-Fisher Scientific, Waltham, MA, USA); a water-soluble tetrazolium salt (WST)-8-based cell viability assay kit (DoGen, Seoul, Korea); bovine serum albumin (GenDEPOT, Katy, TX, USA); secondary horseradish peroxidase (HRP)-conjugated antibodies (GeneTex, Inc., Irvine, CA, USA); *n*-butanol (J.T. Baker, Mexico City, Mexico); berberine, palmatine, coptisine, and jatrorrhizine (Avention, Incheon, Korea).

### 4.3. Fingerprint Analysis of CRE

High-performance liquid chromatography (HPLC) analysis for the simultaneous determination of four reference components in the CRE sample was conducted by using an LC-20A Prominence HPLC system (Shimadzu Co., Kyoto, Japan) equipped with binary pumps, a column oven, an autosampler, and a photodiode array (PDA) detector. In brief, 25 mg of the lyophilized CRE was dissolved in 25 mL of 70% methanol and extracted for 60 min using an ultrasonicator (Branson 8510E-DTH, Danbury, CT, USA), and the extracted solution was then filtered through a 0.2 mm membrane filter (PALL Life Sciences, Ann Arbor, MI, USA). Then, the CRE sample and the four reference components (jatrorrhizine, coptisine, palmatine, and berberine) were subjected to analysis in an HPLC-PDA system. They were separated on a SunFire C18 column (4.6 × 250 mm, 5 mm; Milford, MA, USA) maintained at 30 °C. The mobile phase was eluted with 30 mM ammonium bicarbonate and 0.1% (*v/v*) aqueous triethylamine (A) and acetonitrile (B) in gradient elution mode. The flow rate was 1.0 mL/min with the following linear gradient: 0 to 15 min, 90–75% A and 10–25% B; 15 to 25 min, 75–70% A and 25–30% B; 25 to 40 min, 70–55% A and 30–45% B; 40 to 45 min, 55% A and 45% B; and 45 to 60 min, 55–90% A and 45–10% B. CRE was detected using a photodiode array at 200–500 nm (Figure 1).

### 4.4. Cell Culture Conditions

HCT116 human colon cancer cells were cultured in the RPMI-1640 culture medium supplemented with 10% FBS, 100 U/mL penicillin, and 100 μg/mL streptomycin in a humidified 5% CO_2_ atmosphere at 37 °C in an incubator. To establish the 5-FU-resistant cell line (HCT116/R), parental cells were cultured with increasing concentrations of 5-FU over a period of more than 10 months, and resistance was confirmed using a WST-8 assay [36]. From the initial dose of 5-FU (2 μM), the concentration was gradually increased to 40 μM under conditions of at least an 80% survival rate after 24 h of culture. Before beginning further experiments, the cells were maintained for 1 week in a culture medium without 5-FU.

### 4.5. Cell Viability Assay

Parental HCT116 cells (HCT116/WT) and the corresponding 5-FU-resistant cells (HCT116/R) were seeded at 2 × 10^3^ cells/well into 96-well microplates. To confirm the specificity of resistance, both cell lines were treated with four kinds of anticancer drugs (5, 25, and 50 μM 5-FU, oxaliplatin, paclitaxel, or cisplatin) for 48 h, and cell viability was then determined using a WST-8 assay. To determine the optimized concentration of CRE under 5-FU-resistant conditions, HCT116/R cells were treated with CRE (5, 10, 20, and 40 μg/mL) for 48 h, which showed that 20 μg/mL CRE (a nontoxic dose) was optimal. Consequently, HCT116/R cells were treated simultaneously with 5-FU (5, 25, or 50 μM) and CRE (20 μg/mL) for 48 h. In addition, we compared the anti-5-FU resistance effects of the four main compounds of *C. Rhizoma* (5 μg/mL berberine, palmatine, coptisine, and jatrorrhizine). The absorbance at 450 nm was measured in a UV spectrophotometer (Molecular Devices, San jose, CA, USA).

### 4.6. Real-Time RT-PCR Analysis

To investigate drug resistance mechanisms, we used real-time RT-PCR to analyze the mRNA expression of three major molecules—P-glycoprotein (P-gp), glutathione S-transferase (GST), and thymidylate synthase (TS)—with glyceraldehyde 3-phosphate dehydrogenase (GAPDH) as the control. HCT116/WT or HCT116/R cells were seeded at 3 × 10^5^ cells/well into 60 mm plates and were then treated with CRE (20 μg/mL) or 5-FU (25 μM) for 8 h. Total mRNA of cells was extracted using TRIzol reagent (Molecular Research Center, Cincinnati, OH, USA), and cDNA was then synthesized using a high-capacity cDNA reverse transcription kit (Ambion, Austin, TX, USA). PCR was performed using SYBR Green PCR Master Mix (Applied Biosystems, Foster city, CA, USA) and primers, as described in Table 1. Gene expression data were analyzed using a Rotor gene Q thermal cycler (QIAGEN, Hilden, Germany).

### 4.7. Western Blot Analysis

For Western blot analyses, HCT116/R cells were seeded at 3 × 10^5^ cells/well into 60 mm plates and were then treated with CRE (20 μg/mL), four CRE compounds (5 μg/mL, berberine, palmatine, coptisine, jatrorrhizine) or 5-FU (25 μM) for 48 h. Total protein was extracted using Pro-PrepTM protein extraction solution (iNtRON Biotechnology, Seongnam, Korea), and protein concentrations were determined using a bicinchoninic acid protein assay kit (Sigma-Aldrich, St. Louis, MO, USA). Samples were separated by 12% polyacrylamide gel electrophoresis and transferred to polyvinylidene fluoride (PVDF) membranes. After blocking with 5% skim milk for 1 h, membranes were incubated overnight at 4 °C with primary antibodies specific to the following proteins—TS (1:3000), CDK1 (1:1000), CDK2 (1:1000), CDK4 (1:1000), CDK6 (1:1000), cyclin A (1:1000), cyclin B1 (1:1000), cyclin D1 (1:1000), cyclin E (1:1000), and β-actin (1:2500). After washing, membranes were incubated with an HRP-conjugated anti-rabbit or anti-mouse secondary antibody (1:5000) for 1 h. Bands were visualized with an advanced enhanced chemiluminescence (ECL) kit, and band intensities were analyzed with ImageJ version 1.46 (NIH, Bethesda, MD, USA).

### 4.8. Flow Cytometric Analysis

After treatment of HCT116/R cells (3 × 10^5^ cells/well in a 60 mm plate) with CRE (20 μg/mL) or 5-FU (25 μM) for 48 h, the cells were harvested and suspended in cold PBS containing 3% FBS. The cell suspension was mixed with 70% cold ethanol and incubated overnight at 4 °C. The next day, the cells were resuspended in cold PBS containing 3% FBS and were then stained with propidium iodide (PI, 20 μg/mL) for 5 min. A cell cycle distribution assay was conducted using a FACS system (BD Biosciences, San Jose, CA, USA). Histogram generation and cell cycle analysis were carried out with FlowJo Academic software with a dongle (NIH, Bethesda, MD, USA).

### 4.9. Statistical Analysis

All data are expressed as the mean ± standard deviation (SD) values. Statistical significance was analyzed by one-way analysis of variance (ANOVA) followed by Tukey’s HSD test for post-hoc multiple comparisons using IBM SPSS statistics software, version 20.0 (SPSS Inc., Chicago, IL, USA). Differences were considered statistically significant at *p* < 0.05 or 0.01 as indicated.

## 5. Conclusions

This study first proposed the potential of CRE to overcome 5-FU resistance via the regulation of TS overexpression in CRC. In general, however, there are various mechanisms of drug resistance in tumors, including efflux transporters, drug metabolism, and epigenetic gene alterations in cancer cells [7,36,37,38]. Accordingly, this study has some limitations, and further study is needed to expand the investigations regarding the other anticancer drug resistance patterns mentioned above and various anticancer agents. In addition, further studies are needed to clearly identify the active compounds of CRE that can combat anticancer drug resistance. Taken together, the results of this study indicate the potential of CRE as an anti-chemotherapeutic resistance agent, especially in 5-FU-resistant colorectal tumors. The underlying mechanisms might involve the modulation of TS expression, leading to G0/G1 phase arrest.

## Figures and Tables

**Table 1:** Primer sequences used in this experiment.

Gene Name	Primer Sequence (Forword and Reverse, 5′→3′)
^1^ P-gp	AAG GCC TAA TGC CGA ACA CA TCC AGG CTC AGT CCC TGA AG
^2^ GST	TTC CTG TGG CAT AAT GTG AT CTG ATT CAA AGG CAA ATC TC
^3^ TS	ACC GAG CTC CCG AGA CTT TTT GGA CAG CCT ACC AAG CTT AAG AAT CCT GAG CTT TGG GAA
^4^ GAPDH	CAT GGC CTT CCG TGT TCC T CCT GCT TCA CCA CCT TCT TGA

^1^ permeability-glycoprotein; ^2^ glutathione *S*-transferase; ^3^ thymidylate synthase; ^4^ glyceraldehyde 3-phosphate dehydrogenase.

## Data Availability

The data that support the findings of this study are available from the corresponding author, [Son, C.-G], upon reasonable request.

## Supplementary Materials

The following are available online. Figure S1: LDH concentration in cell supernatant and lysate of HCT116/R cells.

## Sample Availability

Sample of the *coptidis rhizoma* extract is available from the corresponding author, [Son, C.-G], upon reasonable request.

## References

[1] Sung H., Ferlay J., Siegel R.L., Laversanne M., Soerjomataram I., Jemal A., Bray F. (2021). Global cancer statistics 2020: GLOBOCAN estimates of incidence and mortality worldwide for 36 cancers in 185 countries. CA Cancer J. Clin..

[2] Karsa L., Lignini T., Patnick J., Lambert R., Sauvaget C. (2010). The dimensions of the CRC problem. Best Pr. Res. Clin. Gastroenterol..

[3] Nizioł M., Kostrzewska B., Kamińska D., Domurat M., Zińczuk J., Misiura M., Guzińska-Ustymowicz K., Pryczynicz A. (2019). Symptoms of colorectal cancer contributes to its localization and advancement. Prog. Health Sci..

[4] Lee G.-Y., Lee J.-S., Son C.-G., Lee N.-H. (2020). Combating Drug Resistance in Colorectal Cancer Using Herbal Medicines. Chin. J. Integr. Med..

[5] Popat S., Matakidou A., Houlston R.S. (2004). Thymidylate Synthase Expression and Prognosis in Colorectal Cancer: A Systematic Review and Meta-Analysis. J. Clin. Oncol..

[6] Grothey A., Sargent D. (2005). Overall survival of patients with advanced colorectal cancer correlates with availability of fluor-ouracil, irinotecan, and oxaliplatin regardless of whether doublet or single-agent therapy is used first line. J. Clin. Oncol..

[7] Abdel-Rahman O., Karachiwala H. (2019). Impact of age on toxicity and efficacy of 5-FU-based combination chemotherapy among patients with metastatic colorectal cancer; a pooled analysis of five randomized trials. Int. J. Color. Dis..

[8] Healey E., Stillfried G.E., Eckermann S., Dawber J.P., Clingan P.R., Ranson M. (2013). Comparative effectiveness of 5-fluorouracil with and without oxaliplatin in the treatment of colorectal cancer in clinical practice. Anticancer. Res..

[9] Folprecht G., Cunningham D., Glimelius B., Dicostanzo F., Wils J., Scheithauer W., Rougier P., Aranda E., Pabst U., Köhne C. (2004). Efficacy of bolus and infusional 5-FU in elderly and non-elderly patients with metastatic colorectal cancer-a pooled analysis of clinical trials. J. Clin. Oncol..

[10] He L., Zhu H., Zhou S., Wu T., Wu H., Yang H., Mao H., SekharKathera C., Janardhan A., Edick A.M. (2018). Wnt pathway is involved in 5-FU drug resistance of colorectal cancer cells. Exp. Mol. Med..

[11] André T., De Gramont A.A., Vernerey D., Chibaudel B.B., Bonnetain F., Tijeras-Raballand A.A., Scriva A.A., Hickish T.T., Tabernero J., Van Laethem J.L. (2015). Adjuvant Fluorouracil, Leucovorin, and Oxaliplatin in Stage II to III Colon Cancer: Updated 10-Year Survival and Outcomes According to BRAF Mutation and Mismatch Repair Status of the MOSAIC Study. J. Clin. Oncol..

[12] Francipane M.G., Bulanin D., Lagasse E. (2019). Establishment and Characterization of 5-Fluorouracil-Resistant Human Colorectal Cancer Stem-Like Cells: Tumor Dynamics under Selection Pressure. Int. J. Mol. Sci..

[13] Thomas S.A., Grami Z., Mehta S., Patel K. (2016). Adverse Effects of 5-fluorouracil: Focus on Rare Side Effects. Cancer Cell Microenviron..

[14] Röhrl K., Guren M.G., Småstuen M.C., Rustøen T. (2019). Symptoms during chemotherapy in colorectal cancer patients. Support. Care Cancer.

[15] Xiong L.-R., Jing P.-W., Song X.-Y., Wang Y.-P., Wang L. (2017). 5-FU-Injured Bone Marrow Stromal Cells Initiate Stress-induced Premature Senescence of Hematopoietic Cells. Zhongguo Shi Yan Xue Ye Xue Za Zhi.

[16] Colucci G., Gebbia V., Paoletti G., Giuliani F., Caruso M., Gebbia N., Cartenì G., Agostara B., Pezzella G., Manzione L. (2005). Phase III Randomized Trial of FOLFIRI Versus FOLFOX4 in the Treatment of Advanced Colorectal Cancer: A Multicenter Study of the Gruppo Oncologico Dell’Italia Meridionale. J. Clin. Oncol..

[17] Sui H., Liu X., Jin B.-H., Pan S.-F., Zhou L.-H., Yu N.A., Wu J., Cai J.-F., Fan Z.-Z., Zhu H.-R. (2013). Zuo Jin Wan, a Traditional Chinese Herbal Formula, Reverses P-gp-Mediated MDRIn VitroandIn Vivo. Evid.-Based Complement. Altern. Med..

[18] Wang J., Wang L., Lou G.-H., Zeng H.-R., Hu J., Huang Q.-W., Peng W., Yang X.-B. (2019). Coptidis Rhizoma: A comprehensive review of its traditional uses, botany, phytochemistry, pharmacology and toxicology. Pharm. Biol..

[19] Huang T., Xiao Y., Yi L., Li L., Wang M., Tian C., Ma H., He K., Wang Y., Han B. (2017). Coptisine from Rhizoma Coptidis Suppresses HCT-116 Cells-related Tumor Growth in vitro and in vivo. Sci. Rep..

[20] Wang W. (2016). A review on pharmacologic effects of effective ingredients in huanglian. Clin. J. Chin. Med..

[21] Dan L., Guangshang C., Xixi S., Qianqian C., Hongsheng S. (2017). An overview of the antiarrhythmic study of alkaloids in Coptidis Rhizoma. Shandong J. Trad Chin. Med..

[22] Mou S.-J., Yang P.-F., Liu Y.-P., Xu N., Jiang W.-W., Yue W.-J. (2020). BCLAF1 promotes cell proliferation, invasion and drug-resistance though targeting lncRNA NEAT1 in hepatocellular carcinoma. Life Sci..

[23] Satapathy S.R., Sjölander A. (2020). Cysteinyl leukotriene receptor 1 promotes 5-fluorouracil resistance and resistance-derived stemness in colon cancer cells. Cancer Lett..

[24] Das D., Preet R., Mohapatra P., Satapathy S.R., Kundu C.N. (2013). 1,3-Bis(2-chloroethyl)-1-nitrosourea enhances the inhibitory effect of Resveratrol on 5-fluorouracil sensitive/resistant colon cancer cells. World J. Gastroenterol..

[25] Longley D.B., Allen W.L., Johnston P.G. (2006). Drug resistance, predictive markers and pharmacogenomics in colorectal cancer. Biochim. Biophys. Acta (BBA) Bioenerg..

[26] Huang L., Zhang S., Zhou J., Li X. (2019). Effect of resveratrol on drug resistance in colon cancer chemotherapy. RSC Adv..

[27] Housman G., Byler S., Heerboth S., Lapinska K., Longacre M., Snyder N., Sarkar S. (2014). Drug resistance in cancer: An over-view. Cancers.

[28] Yokogawa T., Yano W., Tsukioka S., Osada A., Wakasa T., Ueno H., Hoshino T., Yamamura K., Fujioka A., Fukuoka M. (2021). dUTPase inhibition confers susceptibility to a thymidylate synthase inhibitor in DNA-repair-defective human cancer cells. Cancer Sci..

[29] Showalter S.L., Showalter T.N., Witkiewicz A., Havens R., Kennedy E.P., Hucl T., Kern S.E., Yeo C.J., Brody J.R. (2008). Eval-uating the drug-target relationship between thymidylate synthase expression and tumor response to 5-fluorouracil: Is it time to move forward?. Cancer Biol. Ther..

[30] Chong D., Ma L., Liu F., Zhang Z., Zhao S., Huo Q., Zhang P., Zheng H., Liu H. (2017). Synergistic antitumor effect of 3-bromopyruvate and 5-fluorouracil against human colorectal cancer through cell cycle arrest and induction of apoptosis. Anti-Cancer Drugs.

[31] Otto T., Sicinski P. (2017). Cell cycle proteins as promising targets in cancer therapy. Nat. Rev. Cancer.

[32] Zhang J., Zhou L., Zhao S., Dicker D.T., El-Deiry W.S. (2017). The CDK4/6 inhibitor palbociclib synergizes with irinotecan to promote colorectal cancer cell death under hypoxia. Cell Cycle.

[33] Chen J., Wang F., Liu J., Lee F.S.-C., Wang X., Yang H. (2008). Analysis of alkaloids in Coptis chinensis Franch by accelerated solvent extraction combined with ultra performance liquid chromatographic analysis with photodiode array and tandem mass spec-trometry detections. Anal. Chim. Acta.

[34] Ma B.-L., Ma Y.-M., Shi R., Wang T.-M., Zhang N., Wang C.-H., Yang Y. (2010). Identification of the toxic constituents in Rhizoma Coptidis. J. Ethnopharmacol..

[35] Yang Y., Zhang Z., Li S., Ye X., Li X., He K. (2014). Synergy effects of herb extracts: Pharmacokinetics and pharmacodynamic basis. Fitoterapia.

[36] Longley D.B., Johnston P.G. (2005). Molecular mechanisms of drug resistance. J. Pathol..

[37] Zahreddine H., Borden K.L.B. (2013). Mechanisms and insights into drug resistance in cancer. Front. Pharmacol..

[38] Blondy S., David V., Verdier M., Mathonnet M., Perraud A., Christou N. (2020). 5-Fluorouracil resistance mechanisms in colorectal cancer: From classical pathways to promising processes. Cancer Sci..

