# Supplementary material for: Coptidis Rhizoma Extract Reverses 5-Fluorouracil Resistance in HCT116 Human Colorectal Cancer Cells via Modulation of Thymidylate Synthase
Source: Molecules. 2021 Mar 25;26(7):1856. doi: 10.3390/molecules26071856 (PMC8036817; doi:10.3390/molecules26071856)
Supplement: Supplementary file 1 [file molecules-26-01856-s001.pdf]

Supplementary Information

Results

Evaluation of cell death and TS overexpression-derived 5-FU resistance

Cotreatment with CRE (20 µg/mL) and 5-FU (25 µM) did not induce the release of intracellular LDH in HCT116/R cells, and it was similar by 5-FU or CRE treatment alone (Fig. S1A). Dose-dependently significant resistance to Raltitrexed, TS inhibitor, was observed in HCT116/R cells compared to HCT116/WT cells (Fig. S1B).

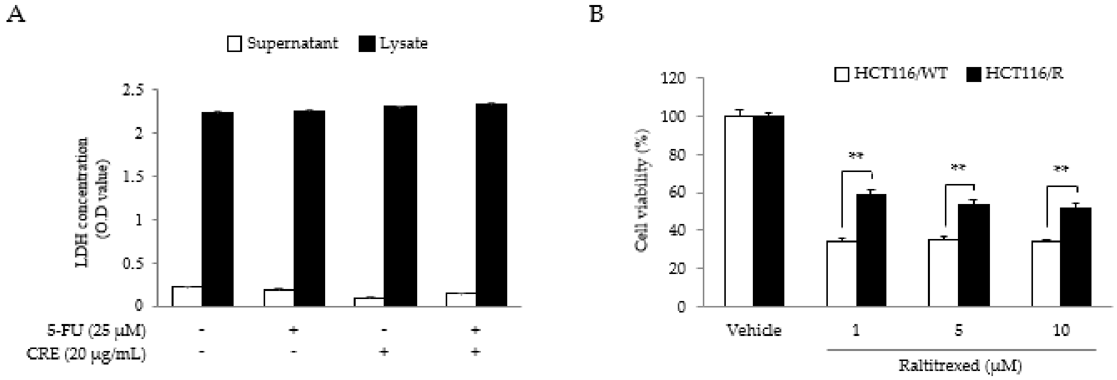

Figure S1. LDH concentration in cell supernatant and lysate of HCT116/R cells. LDH was measured by cotreatment or single-treatment with 5-FU and/or CRE (A). Cell viability was measured to investigate anticancer drug resistance against Raltitrexed in both HCT116/WT and HCT116/R cells (B).

Material method

LDH assay

HCT116/R cells were seeded at 2 × 10^3 cells/well into 96-well microplates. After incubation for 12 h, cells were treated with CRE (20 µg/mL) and/or 5-FU (25 µM) for 48 h. To evaluate cell death, LDH concentration was determined using a LDH assay (EZ-LDH, DoGen, Republic of Korea). The absorbance at 450 nm was measured in a UV spectrophotometer (Molecular Devices, CA, USA).

Cell viability assay

HCT116/R cells were seeded at 2 × 10^3 cells/well into 96-well microplates. To confirm the TS overexpression-derived 5-FU-resistant mechanisms, HCT116/R cells were treated with Raltitrexed (1, 5, and 10 µM) for 48 h, and then cell viability was determined using a WST-8 assay (EZ-Cytox, DoGen, Republic of Korea). The absorbance at 450 nm was measured in a UV spectrophotometer (Molecular Devices, CA, USA).
